# N-Acetylcysteine Reverses Antiretroviral-Mediated Microglial Activation by Attenuating Autophagy-Lysosomal Dysfunction

**DOI:** 10.3389/fneur.2020.00840

**Published:** 2020-09-04

**Authors:** Ashutosh Tripathi, Annadurai Thangaraj, Ernest T. Chivero, Palsamy Periyasamy, Maria E. Burkovetskaya, Fang Niu, Ming-Lei Guo, Shilpa Buch

**Affiliations:** Department of Pharmacology and Experimental Neuroscience, University of Nebraska Medical Center, Omaha, NE, United States

**Keywords:** combined antiretroviral therapy, N-acetylcysteine, lysosome, autophagy, microglial activation, neuroinflammation

## Abstract

Successful suppression of viral replication by combined antiretroviral therapy (cART) in HIV-1 infected individuals is paradoxically also accompanied by an increased prevalence of HIV-associated neurocognitive disorders (HAND) in these individuals. HAND is characterized by a state of chronic oxidative stress and inflammation. Microglia are extremely sensitive to a plethora of stimuli, including viral proteins and cART. The current study aimed to assess the effects of cART-mediated oxidative stress on the induction of inflammatory responses in microglia. In the present study, we chose a combination of three commonly used antiretroviral drugs—tenofovir disoproxil fumarate, emtricitabine, and dolutegravir. We demonstrated that exposure of microglia to the chosen cART cocktail induced generation of reactive oxygen species, subsequently leading to lysosomal dysfunction and dysregulated autophagy, ultimately resulting in the activation of microglia. Intriguingly, the potent antioxidant, N-acetylcysteine, reversed the damaging effects of cART. These *in vitro* findings were further corroborated *in vivo* wherein cART-treated HIV transgenic (Tg) rats demonstrated increased microglial activation, exaggerated lysosome impairment, and dysregulated autophagy in the prefrontal cortices compared with HIV Tg rats not exposed to cART. Similar to *in vitro* findings, the treatment of HIV Tg rats with N-acetylcysteine also mitigated the deleterious effects of cART. Taken together, our findings suggest that oxidative stress-mediated lysosomal dysfunction plays a critical role in the pathogenesis of HAND in drug-treated HIV-infected individuals and that antioxidant-mediated mitigation of oxidative stress could thus be considered as an adjunctive therapeutic strategy for ameliorating/dampening some of the neurological complications of HAND.

## Introduction

With the advent of combined antiretroviral therapy (cART), HIV infection has transformed from a death sentence to a more chronic and manageable disease (1–3). Almost 50% of the infected individuals develop HIV-associated neurocognitive disorders (HANDs), with symptoms ranging from asymptomatic to mild cognitive–motor disorders. This, in turn, severely impacts the quality of life of those afflicted with the disease (3). Although the mechanism(s) underlying pathogenesis of HAND is not clearly understood, associated oxidative stress, immune activation, and inflammation have been implicated in the process (4, 5).

Microglia are the predominant brain-resident macrophages that maintain central nervous system (CNS) homeostasis under basal conditions and also during moderate activation. Prolonged microglial activation, on the other hand, impairs the ability of microglia to maintain cellular homeostasis and leads to significant neuronal dysfunction and cognitive impairment, with exacerbated neuroinflammation in the CNS (6, 7). Exacerbated microglial activation and neuroinflammation are hallmark features of HAND pathogenesis in HIV-infected individuals on cART (8–10). Prolonged exposure to cART has been reported to activate microglia (11–13). Impaired lysosomal functioning has been shown to underlie microglial activation and increased neuroinflammation (14, 15). In fact, findings from our group have identified the role of lysosomal dysfunction in cART-mediated dysregulation of autophagy (13).

Lysosomes are specialized membrane-enclosed cellular organelles that receive and degrade macromolecules from phagocytosis, endocytosis, or autophagy pathways (16). Lysosomes are extremely sensitive to oxidative stress, which causes lysosomal dysfunction (17). The role of oxidative stress, caused by an imbalance between the production and elimination of reactive oxygen species (ROS), has been well-documented in the onset of chronic inflammatory diseases (18, 19). Along these lines, redox imbalance has been reported in the serum and cerebrospinal fluid of HIV-1 patients treated with cART (20, 21). In fact, there is also a report on the involvement of oxidative stress in antiretroviral drugs-mediated neuronal damage in the CNS (22). Oxidative stress could thus play a key role in the pathogenesis of HAND. Strategies aimed at reducing or preventing the generation of oxidative stress (23) could thus be a plausible mechanism to mitigate cART-mediated induction of microglial activation and neuroinflammation.

In the current study, induction of oxidative stress was examined by assessing the levels of ROS in microglial cells exposed to cART. Furthermore, it was also shown that the treatment of microglia with potent antioxidant N-acetylcysteine (NAC) abrogated cART-mediated activation of microglia. We acknowledge that there are several other combinations of antiretroviral drugs, and the most common first-line cART regimens include two nucleoside reverse transcriptase inhibitors (NRTIs) plus a boosted protease inhibitor or an integrase inhibitor (24, 25). In the present study, we chose to study a combination of two NRTIs, tenofovir disoproxil fumarate (TDF) and emtricitabine (FTC), and an integrase inhibitor, dolutegravir (DTG). This combination has been effectively used in the simian immunodeficiency virus (SIV)/macaque model (26, 27), and these drugs are also common in the clinical setting (25, 28–30). Our *in vitro* findings were further corroborated by *in vivo* studies, wherein HIV Tg rats (expressing seven of the nine HIV proteins) that were treated with cART exhibited exaggerated neuroinflammation and lysosomal damage compared with HIV Tg rats not treated with cART. Consistently, the treatment of HIV Tg rats with NAC resulted in the failure of cART to mediate microglial activation, and this was also accompanied by the restoration of impaired lysosomal and autophagy processes. Our findings thus suggest that oxidative stress-mediated lysosomal dysfunction likely plays a critical role in the pathogenesis of HAND in cART-treated HIV-infected individuals and that antioxidant-mediated mitigation of oxidative stress could be considered as an adjunctive therapeutic strategy for ameliorating/dampening some of the neurological complications of HAND.

## Materials and Methods

### Reagents

Antiretroviral drugs TDF, FTC (Gilead Sciences, Foster City, CA, USA), and DTG (ViiV Healthcare, Research Triangle Park, NC, USA) were used. NAC (A7250) and (2-(2,2,6,6-Tetramethylpiperidin-1-oxyl-4-ylamino)-2-oxoethyl)triphenylphosphonium chloride (mitoTEMPO; SML0737) were purchased from Sigma-Aldrich, St. Louis, MO, USA, and 4-hydroxy-2,2,6,6-tetramethylpiperidin-1-oxyl (TEMPOL; sc-200825) was purchased from Santa Cruz Biotechnology, Dallas, TX, USA. Antibody resources: beclin 1 (BECN1; sc-11427) was purchased from Santa Cruz Biotechnology, Dallas, TX, USA. Lysosome associated membrane protein 2 (LAMP2; NB300-591), microtubule-associated protein 1 light chain 3 beta (MAP1LC3B; NB100-2220), and integrin subunit alpha M (ITGAM; NB110-89474) were purchased from Novus Biological Company, Centennial, CO, USA. Cathepsin D (CTSD; ab75852) and caspase 3 (CASP3; ab13585) were purchased from Abcam, Cambridge, MA, USA. Galectin 3 (GAL3) (A3A12) was purchased from Invitrogen. Sequestosome 1 (SQSTM1; PM045) was purchased from MBL International, Woburn, MA, USA, and allograft inflammatory factor 1 (AIF1; 019-19741) was purchased from Wako Pure Chemicals Industries, Chuo-ku, Osaka, Japan. Goat anti-rabbit (sc-2004) and goat anti-mouse (sc-2005) were purchased from Santa Cruz Biotechnology, Dallas, TX, USA.

### Animals

Male Sprague–Dawley (8–9 months old) HIV-1 transgenic rats (HIV-1, F344) were housed in a 12-h light and 12-h dark cycle under conditions of constant temperature and humidity. Food and water were available *ad libitum*. All animal procedures were performed according to the protocols approved by the Institutional Animal Care and Use Committee of the University of Nebraska Medical Center and the National Institutes of Health.

### Rat Primary Microglial Cell Isolation

Primary microglial cells were obtained from the cerebral cortices of 1–3-days-old newborn Sprague–Dawley pups. Briefly, cerebral cortices from the pups were dissected, and meninges were removed. Mixed glial cultures were prepared by form-dissected brain cortices after digestion and dissociation in Hank's buffered salt solution (HBSS, Thermo Fisher Scientific Waltham, MA, USA, 14025076) supplemented with 0.25% trypsin (Thermo Fisher Scientific, Waltham, MA, USA, 25300-054), followed by triturating and passing through a 40-μm nylon mesh. Cells were centrifuged and resuspended in Dulbecco modified Eagle medium (DMEM, Thermo Fisher Scientific, Waltham, MA, USA, 11995-065) supplemented with 10% heated inactivated fetal bovine serum (FBS, Thermo Fisher Scientific Waltham, MA, USA, 16000-044), 100-U/ml penicillin−0.1-mg/ml streptomycin, and 0.25-ng/ml macrophage colony-stimulating factor (Thermo Fisher Scientific Waltham, MA, USA, PHC9504). Cells were plated at 20 × 10^6^ cells/flask density onto 75-cm^2^ cell culture flasks. The cell culture medium was changed every third day. When confluent (around 8–10 days), mixed glial cultures were shaken at 37°C at 220 g for 2 h, to detach microglia from the flasks. Detached microglia were plated and cultured for an additional 24 h for further experimental use. Microglial purity was evaluated by immune-histochemical staining using the antibody specific for AIF1 (Wako Pure Chemical Industries Chuo-ku, Osaka, Japan, 019-19741) and was >95% pure.

### Antiretroviral Drugs

For *in vitro* experiments, antiretroviral stock solutions were prepared by dissolving the drugs (TDF, FTC, and DTG) in dimethyl sulfoxide (DMSO). Final concentrations of each antiretroviral drugs (TDF, FTC, and DTG) in the cART cocktail were 5 μM. DMSO was <0.01% (v/v) in the cART-treated and control groups.

### Western Blotting

Treated rat primary microglial cells (rPMs) and brain tissues were lysed using radioimmunoprecipitation assay buffer supplemented with a cocktail of protease inhibitor (Thermo Fisher Scientific Waltham, MA, USA, 78429) and phosphatase inhibitor (Thermo Fisher Scientific Waltham, MA, USA, 78426) using Fisherbrand™ Q125 Sonicator. Cell and tissue lysates were centrifuged at 12,000 g for 15 min at 4°C to remove the debris. The concentration of protein was determined by a bicinchoninic acid assay (Thermo Fisher Scientific Waltham, MA, USA, 23227) according to the manufacturer's guidelines. Proteins were electrophoresed in equal concentration in a sodium dodecyl sulfate-polyacrylamide gel under reducing conditions and then transferred to polyvinylidene difluoride membranes (Sigma-Aldrich, St. Louis, MO, USA, IPVH00010). Then, the polyvinylidene difluoride membranes were blocked with 5% non-fat dry milk in 1× Tween–Tris-buffered saline (1.21-g Tris [Fisher Scientific, Hampton, NH, USA, BP152-5], 8.77-g sodium chloride [Fisher Scientific, Hampton, NH, USA, BP358-212], 500-μl Tween-20 [Fisher Scientific, Hampton, NH, USA, BP337-500], pH 7.6 for 1 L). After, blocking blots were then probed with primary antibodies overnight at 4°C. Actin beta (ACTB; Sigma-Aldrich, St. Louis, MO, USA, A5441) was used to normalize the protein. The horseradish peroxidase-conjugated secondary antibodies to goat anti-mouse/rabbit IgG were probed for 1 h followed by detection of immunoreactive bands using Super Signal West Pico Chemiluminescent Substrate (Thermo Fisher Scientific Waltham, MA, USA, 34078).

### Immunocytochemistry

After the treatment, rPMs were fixed with 4% paraformaldehyde at room temperature for 15 min, then permeabilized with 0.3% Triton X-100 (Fisher Scientific, Hampton, NH, USA, BP151-500) in phosphate-buffered saline (PBS, Fisher Scientific, Hampton, NH, USA, SH3025801). Cells were then blocked with 10% normal goat serum in PBS for 1 h at room temperature. Then, primary antibodies were added and incubated overnight at 4°C. Following this, the secondary antibodies were added for 2 h. Cells were then washed in PBS (three times) and mounted with Prolong gold antifade reagent with 4,6-diamidino-2-phenylindole (Thermo Fisher Scientific, Waltham, MA, USA, P36935). Images of fluorescent cells were taken with a Zeiss Observer using a Z1 inverted microscope (Carl Zeiss, Thornwood, NY, USA), and analysis of the images was done using the AxioVs 40 Version 4.8.0.0 software (Carl Zeiss, Thornwood, NY, USA).

### Quantification of Microtubule-Associated Protein 1 Light Chain 3 Beta and Lysosome Associated Membrane Protein 2 Puncta

Fluorescence images were acquired with Zeiss Observer using a Z1-inverted microscope (Carl Zeiss, Thornwood, NY, USA). Images thus acquired were analyzed using Image J software. Firstly, the region of interest in the cells to be analyzed was selected using the polygon selection tool. Fluorescence was converted to black pixels over a white background. The regions of interest were then analyzed by Measure Particle algorithm to record puncta number, area, and size (13).

### Reactive Oxygen Species Detection

Detection of ROS was performed according to the manufacturer's (Life technologies, D-339) recommended protocol using Image-iT™ LIVE Green ROS Detection Kit (Thermo Fisher Scientific, Waltham, MA, USA, 136007). Briefly, cells were seeded in 96 wells a day before the experiment. Cells were washed with HBSS (Thermo Fisher Scientific Waltham, MA, USA, 14025076), supplemented with a 25-μM carboxy-H_2_DCFDA working solution, and incubated for 30 min at 37°C. In addition to carboxy-H_2_DCFDA, the kit provides the common inducer of ROS production tert-butyl hydroperoxide (TBHP) as a positive control. The changes in fluorescence were measured using a spectrofluorometer set at 485-nm excitation and 530-nm emission.

### Real-Time Quantitative PCR

Total RNA was extracted using Quick-RNA™ Miniprep Kit (Zymo Research, Irvine, CA, USA, R1055). Column purified, total RNA was reverse transcribed to complementary DNA using a Verso complementary DNA Synthesis Kit (Thermo Fisher Scientific, Waltham, MA, USA, AB-1453/B). Reverse-transcribed RNA was analyzed by Applied Biosystems™ 7500 Real-Time PCR (Thermo Fisher Scientific, Waltham, MA, USA) using the real-time PCR that was performed using the TaqMan-Master mix and TaqMan gene expression assays with FAM-labeled probes using standard amplification protocol (Applied Biosystems). Rat primers for tumor necrosis factor (*Tnf; Rn01525859_g1)*, interleukin 6 (*Il6*; Rn01410330_m1), interleukin 1 beta (*Il1*β; Rn00580432_m1), and *Gapdh* (Rn01775763_g1) were purchased from Thermo Fisher Scientific, Waltham, MA, USA. *Gapdh* was used as an internal control for normalization. Experiments were carried out in triplicate. The fold change in expression was then obtained by the 2^−ΔΔCT^ method.

### Immunohistochemistry

Animals were perfused, and immunohistochemical procedures were performed as described later. Rapidly frozen sections with 20 μM were co-incubated with primary anti-AIF1 antibody (Wako Pure Chemical Industries, Chuo-ku, Osaka, Japan, 019-19741) and anti-LAMP2 antibody (Santa Cruz Biotechnology, Dallas, TX, USA, sc-19991) overnight at 4°C. Secondary AlexaFluor 488 goat anti-rabbit IgG (A-11008) or AlexaFluor 594 goat anti-mouse (A-11032) from Thermo Fisher Scientific Waltham, MA, USA, was added for 2 h to detect Iba1 and LAMP2, followed by mounting of sections with prolong gold antifade reagent with 4,6-diamidino-2-phenylindole (Thermo Fisher Scientific, Waltham, MA, USA, P36935). Fluorescent images were acquired on a Zeiss Observer. AxioVs 40 4.8.0.0 software (Carl Zeiss, Thornwood, NY, USA) was used to process the images.

### Cathepsin D Activity

CTSD Activity Assay Kit (Fluorometric; ab65302) from Abcam, Cambridge, MA, USA, was used to study CTSD activity. The cell lysates were incubated with reaction buffer for 1 h at 37°C. CTSD activity was determined by comparing the relative fluorescence units (Ex/Em = 328/460 nm) against the levels of the controls.

### Lysosomal Membrane Permeability Assay

Acridine orange is a versatile fluorescence dye that easily crosses the cell membrane and reversibly accumulates into acidified membrane-bound compartments, such as lysosomes. Acridine orange gives fluorescence emission in a concentration-dependent manner, which is red at high concentrations (e.g., in lysosomes) to green at low concentrations (e.g., in the cytosol), with yellow as intermediate (e.g., upon trapping in nucleoli). The ratio of red-to-green emission in comparison with controls may thus either monitor lysosomal leakage or change in lysosomal pH. rPMs were grown in 96-well culture plates. rPMs were first exposed with acridine orange (5 μg/ml) at 37°C for 15 min, which were rinsed, then incubated in HBSS with or without cART and NAC for the indicated times. Cells were examined at 1 h intervals using a Synergy™ Mx Monochromator-Based Multi-Mode Microplate Reader (BioTek Instruments, Inc. Winooski, VT, USA) with excitation wavelength at 485 nm and emission recorded at 530 and 620 nm. To further confirm cART-mediated lysosomal membrane permeabilization (LMP), cells were stained with GAL3 and LAMP2.

### Plasmid Transfection

To study the autophagosome formation and maturation, rPMs were transfected with tandem fluorescent-tagged MAP1LC3B plasmid (ptfLC3; a gift from Tamotsu Yoshimori; Addgene, 21074) (31). Briefly, cells were grown in 10% FBS-DMEM overnight until 70% confluence. Then, the culture medium was replaced with 250 μl of Opti-MEM® I Reduced Serum Medium. Cells were then transfected with the GFP-MAP1LC3B plasmid using Lipofectamine® 3000 Reagent, according to the manufacturer's protocol, for 12 h. After that, the culture medium was replaced with the 10% heat-inactivated FBS-DMEM for 24 h. Cells were then treated with various reagents for the indicated time and processed for further analysis.

### Combined Antiretroviral Therapy Injection in HIV Tg Rats

HIV-1 Tg rats (8–9 months old) were randomly separated into four groups (*n* = 3): (1) saline, (2) cART, (3) cART + NAC, and (4) NAC. For the usage of cART, the preformulated cocktail contained two reverse transcriptase inhibitors, 10-mg/kg TDF, and 25-mg/kg FTC plus 1.25 mg/kg of the integrase inhibitor DTG in a solvent containing 25% (v/v) polyethylene glycol 400 (PEG-400, Sigma-Aldrich, St. Louis, MO, USA, PX1286B-2), 15% (w/v) captisol, and 0.075-N sodium hydroxide (Sigma-Aldrich, St. Louis, MO, USA, S8045) in water. We do acknowledge that there are several combinations of antiretroviral drugs and that the common first-line cART regimen includes two NRTIs and a boosted protease inhibitor or an integrase inhibitor (24, 25). In the present study, we sought to assess the effect of a combination of two NRTIs, TDF and FTC, and an integrase inhibitor, DTG, on microglial activation. The rationale for using the antiretroviral cocktail in this study is based on the fact that this regimen is not only shown to be effective in inhibiting virus replication in the clinical setting (25, 28–30) but also have been efficacious in the SIV–macaque model (26, 27), as well as the humanized mouse model of HIV-1 infection (32). According to the US Food and Drug Administration, due to the increased metabolic rates exhibited by rats, the recommended equivalent drug dose should be ~6 times higher than the human dose (33, 34). We have followed guidelines for maximum injection volume by species and site location (33). The antiretroviral regimen (TDF, FTC, and DTG) and NAC (200 mg/kg) were injected (intraperitoneal) for 3 weeks. Rats were killed 1 h after the last injection. Rats were perfused with 0.1-M cold PBS for brain removal. The prefrontal cortex was used for total RNA and protein extraction along with the brain section preparation.

### Statistical Analysis

The results are presented as mean ± SEM and were evaluated using a one-way analysis of variance followed by a Bonferroni (Dunn) comparison of multiple experimental groups. The Student *t*-test was used for comparing two groups. All the statistical analyses were assessed using the GraphPad Prism software (Version 6.01). Values were considered statistically significant when *P* < 0.05.

## Results

### Combined Antiretroviral Therapy-Mediated Generation of Reactive Oxygen Species in Rat Primary Microglial Cells

**Figure 1:**
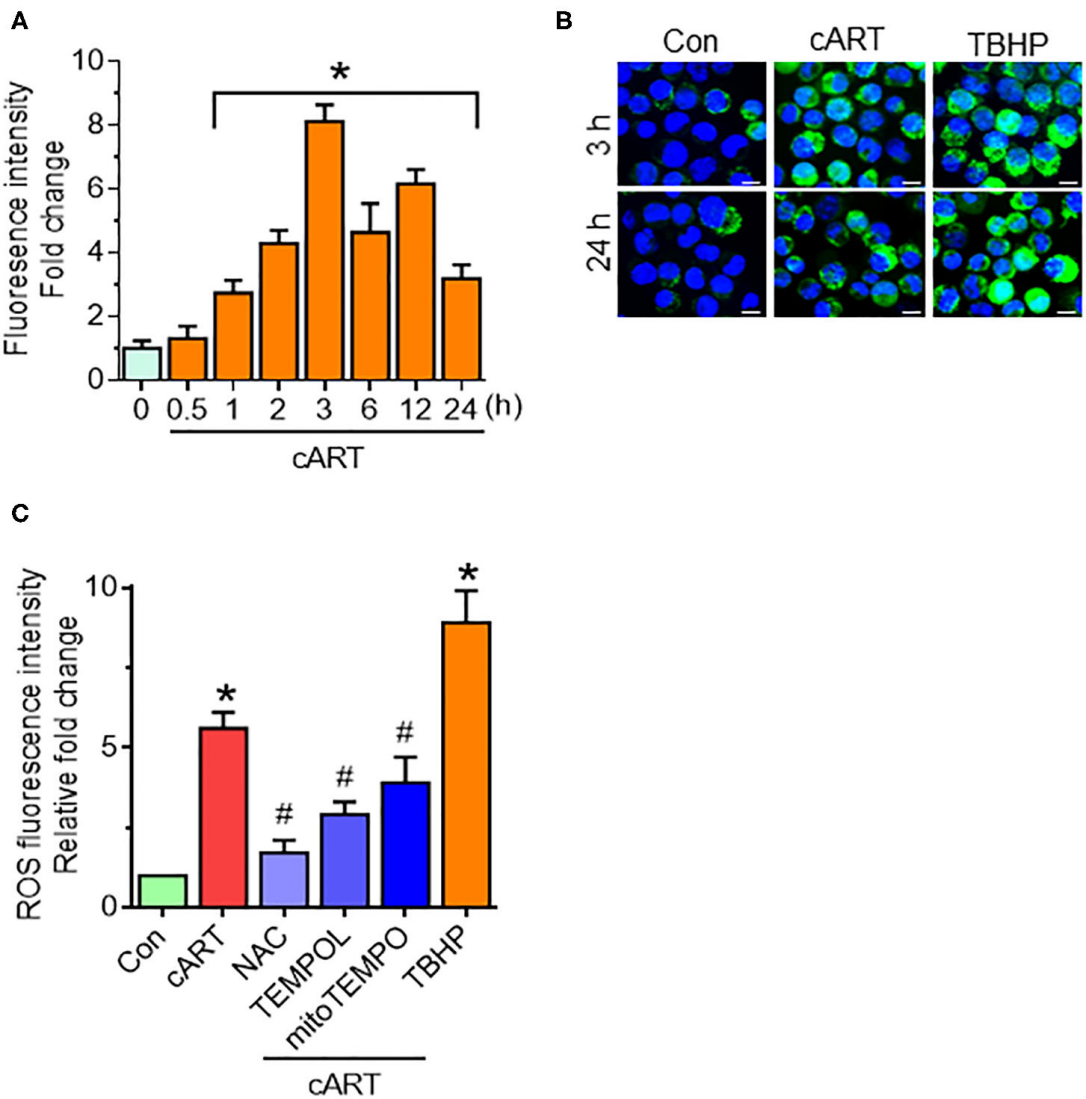
Combined antiretroviral therapy (cART)-mediated reactive oxygen species (ROS) generation in rat primary microglial cells (rPMs). **(A)** Representative graph showing increased generation of ROS in rPMs exposed to cART at varying time points. **(B)** Representative fluorescent-microscopic image showing cART-mediated ROS generation at 3 and 24 h (Scale bar: 10 μm). **(C)** Representative graph showing effect of ROS scavengers N-acetylcysteine (NAC), 4-hydroxy-2,2,6,6-tetramethylpiperidin-1-oxyl (TEMPOL) or (2-(2,2,6,6-Tetramethylpiperidin-1-oxyl-4-ylamino)-2-oxoethyl)triphenylphosphonium chloride (mitoTEMPO) on cART-mediated upregulation of ROS. rPMS was treated with ROS scavengers for 1 h, followed by exposure of cells to cART for an additional 24 h. Tert-butyl hydroperoxide (TBHP) was used as a positive control for ROS generation. Data are from three independent experiments and are represented as means ± SEM using a one-way analysis of variance followed by a Bonferroni (Dunn) comparison of groups. *P < 0.05 vs. control; ^#^*P* < 0.05 vs. cART.

Our recent findings have shown that cART-mediated lysosomal dysfunction causes dysregulated autophagy leading, in turn, to microglial activation (13). In the present study, we sought to explore the upstream mediators underlying these processes. Induction of oxidative stress has been well-documented in the setting of HIV-1 infection and the context of antiretroviral therapy (20, 21, 35). For example, increased levels of oxidants and a concomitant decrease in antioxidant levels have been documented in the serum of HIV-1 infected individuals receiving antiretroviral therapy (21). We thus next sought to determine the possible involvement of ROS in cART-mediated lysosomal dysfunction and autophagy dysregulation. rPMs were exposed to cART (TDF, FTC, and DTG), each at 5 μM for various time points (0–24 h), and assessed for a generation of ROS using the DCFH-DA assay. The rationale for choosing the combination and concentrations of drugs is based on several published reports (36–38). In our previous published study (13), we have studied the effects of individual and the combinations of TDF, FTC, and DTG on lysosomal function. Our data showed a combination of three antiretroviral drugs that significantly affected lysosomal function, which is often recommended as first-line therapy for HIV-1 infection (24). Thus, cART (TDF, FTC, and DTG; each at 5 μM) was chosen for the subsequent experiments. Firstly, the toxicity of the cART cocktail was checked by analyzing cell survival. As shown in Figure S1A, there was no significant difference in the cell survival of the cART-treated and non-treated rPMs. Next, as shown in Figure 1A, exposure of rPMs to cART resulted in significant induction of ROS within 60 min, with a peak induction at 3 h (~8-fold; representative ROS staining is shown in Figure 1B), followed by a gradual decline, however, maintained ~4-fold higher as compared with control rPMs. Next, we inhibited the generation of ROS by treating the rPMs with ROS scavengers, such as NAC (5 mM; a thiol-containing ROS scavenger), TEMPOL (20 μM; a non-thiol-containing ROS scavenger), or mitoTEMPO (10 μM; a mitochondria-specific ROS scavenger), for 1 h followed by exposure of cells to cART for an additional 24 h. TBHP was used as a positive control for ROS generation. As shown in Figure 1C, treatment of the cells with either NAC, TEMPOL, or mitoTEMPO significantly abrogated cART-mediated induction of ROS. Among the three ROS scavengers, NAC showed a maximum ROS scavenging effect in the rPMs treated with cART. NAC is a potent antioxidant that is well-known for its ability to mitigate oxidative stress and its downstream adverse effects (39). NAC exhibits both direct as well as indirect antioxidant properties. Its direct effect is due to a free thiol group interacting with and scavenging ROS. Its indirect antioxidant effect, on the other hand, is related to its role as glutathione (GSH) precursor, leading to increased intracellular GSH concentrations (40). There are reports suggesting replenishment of whole-blood GSH and T-cell GSH levels in HIV-infected individuals after NAC treatment (41).

### N-Acetylcysteine-Reversed Combined Antiretroviral Therapy-Mediated Lysosomal Dysfunction in Rat Primary Microglial Cells

**Figure 2:**
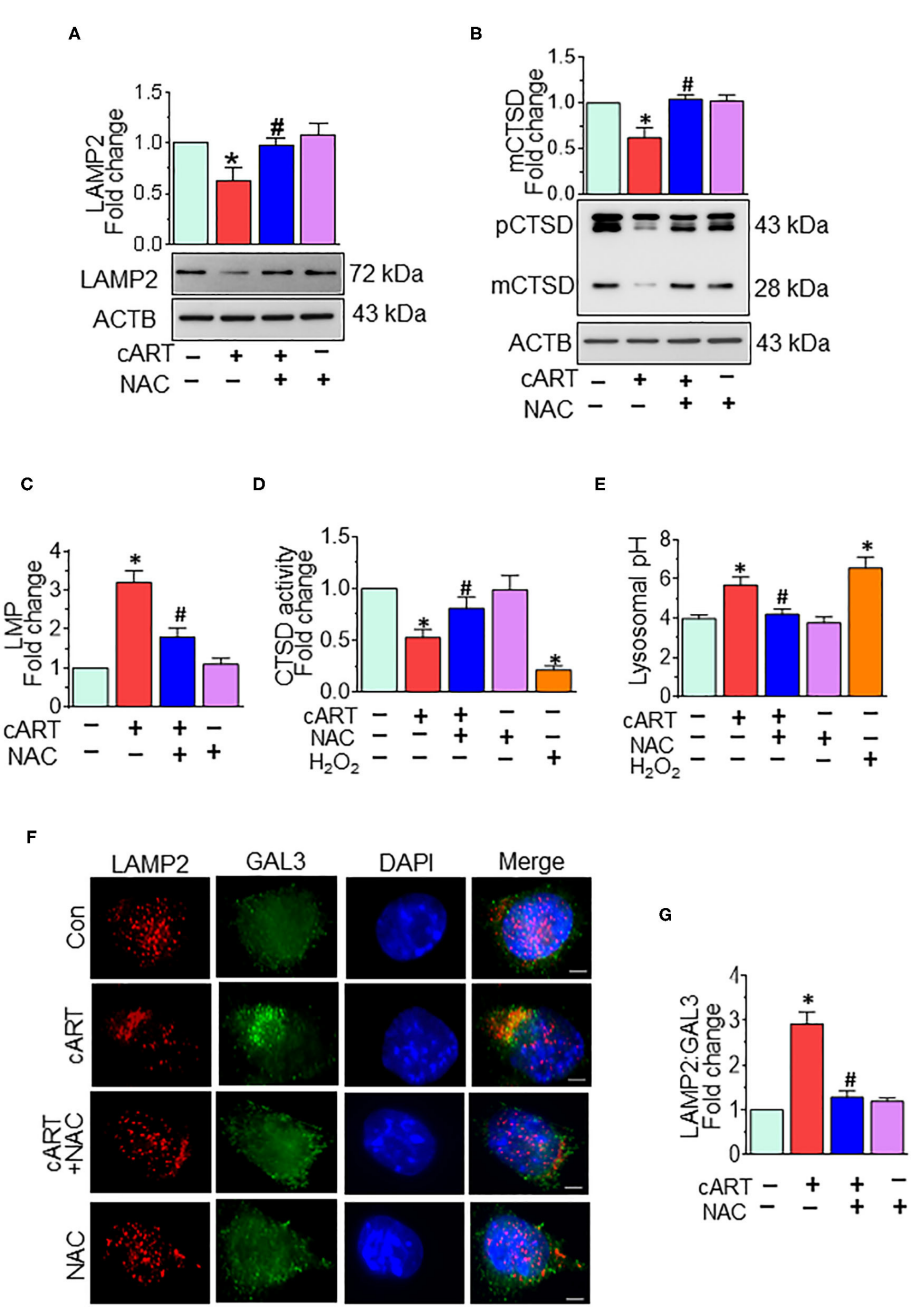
N-acetylcysteine (NAC) reverses combined antiretroviral therapy (cART)-mediated lysosomal dysfunction in rat primary microglial cells (rPMs). rPMs were seeded into six-well plates and subject to various treatments for 24 h. Protein homogenates were prepared for the detection of indicated molecules. **(A,B)** Representative Western blots showing treatment of microglia with NAC-reversed cART-mediated downregulation of lysosomal-associated membrane protein 2 (LAMP2) **(A)** and mature cathepsin D (mCTSD) expression levels **(B)**. mCTSD is the mature form of pro-cathepsin (pCTSD). Maturation of cathepsins and their activity is dependent on the acidity of the lysosomes (low pH). **(C–E)** Representative bar graph showing NAC-mediated protection of lysosomal membrane permeabilization (LMP) **(C)**, CTSD activity **(D)**, and lysosomal pH **(E)** in the presence of cART. **(F)** Representative fluorescent-microscopic image showing the cART-mediated increase in LAMP2 and galectin 3 (GAL3) colocalization (Scale bar: 5 μm). **(G)** Representative bar graph showing quantitative values of LAMP2 and GAL3 colocalization. A minimum of 50 randomly chosen cells for each experimental group were analyzed. For all Western blots, ACTB served as a protein loading control. Data are from three independent experiments and are represented as means ± SEM and were analyzed using one-way ANOVA. *P < 0.05 vs. control; ^#^*P* < 0.05 vs. cART.

Our previous findings showed cART-mediated lysosomal dysfunction (13). Our data showed significant downregulation of LAMP2 expression starting at 6 h, with a continued trend of downregulation up to 24 h in the rPMs exposed to cART. In the present study, we sought to determine whether cART-mediated ROS induction played a role in lysosomal dysfunction. To validate this, rPMs were treated with cART cocktail with or without NAC (5 mM) for 24 h, after which the expression of LAMP2 and mature (m)CTSD proteins was assessed by Western blotting. LAMP2 is a lysosome membrane protein whose downregulation affects lysosomal membrane permeability. mCTSD is the mature form of pro-cathepsin (pCTSD). The maturation of cathepsins and their activity is dependent on the acidity of the lysosomes (low pH) (42–44). Interestingly, rPMs treated with NAC abrogated cART-mediated downregulation of LAMP2 (Figure 2A) and mCTSD (Figure 2B). Additionally, we also performed a lysosomal functional analysis in rPMs treated with cART cocktail with or without NAC for 24 h. Treatment of rPMs with NAC significantly abrogated cART-mediated upregulation of LMP (Figure 2C). Furthermore, NAC abrogated cART-mediated downregulation of CTSD activity (Figure 2D). Next, we thus sought to determine the role of NAC in maintaining lysosomal pH in the rPMs treated with cART. As shown in Figure 2E, the treatment of rPMs with NAC inhibited a cART-mediated increase in lysosomal pH in rPMs.

To further assess the role of cART on LMP, we analyzed the translocation of GAL3 into the damaged lysosomes. GAL3 staining is one of the best approaches for determining LMP, a process by which leaky lysosomes are detected because of abundant and rapid translocation of GAL3 into the leaky lysosomes. Under normal conditions, GAL3 is uniformly distributed in the cells, and any change in LMP results in the translocation of GAL3 into the leaky lysosomes, thereby forming the puncta (45). As shown in Figures 2F,G, exposure of rPMs to cART (24 h) significantly increased the colocalization of LAMP2 with GAL3, and this effect was significantly abrogated in cells that were treated with NAC, followed by exposure to cART. Furthermore, acridine orange staining was performed in rPMs exposed to cART to validate further the findings observed with LMP. Acridine orange is a fluorescent dye that easily traverses the cell membrane. Acridine orange, which is a weak base, reversibly accumulates into the acidified membrane-bound compartments, such as the lysosomes. The fluorescent emission of acridine orange is concentration-dependent, being red at high concentrations (e.g., in lysosomes) and green at low concentrations (e.g., in the cytosol), with yellow as intermediate (e.g., upon trapping in nucleoli). Thus, lysosomal leakage or lysosomal pH change can be easily assessed by determining the shifts from red-to-green emission ratio in comparison with the respective control cells. As shown in Figures S1B,C, rPMs exposed to cART exhibited increased green emission that was significantly abrogated in cells treated with NAC followed by exposure to cART. Furthermore, as the cART stock solutions were prepared in DMSO, the effect of untreated and DMSO (vehicle, 0.01% [v/v]) treatment was checked on rPMs. As shown in Figure S3, there was no significant difference in the ROS, CD11b, and LAMP2 levels in the untreated and DMSO (vehicle)-treated rPMs.

### N-Acetylcysteine-Reversed Combined Antiretroviral Therapy-Mediated Autophagy Dysregulation in Rat Primary Microglial Cells

**Figure 3:**
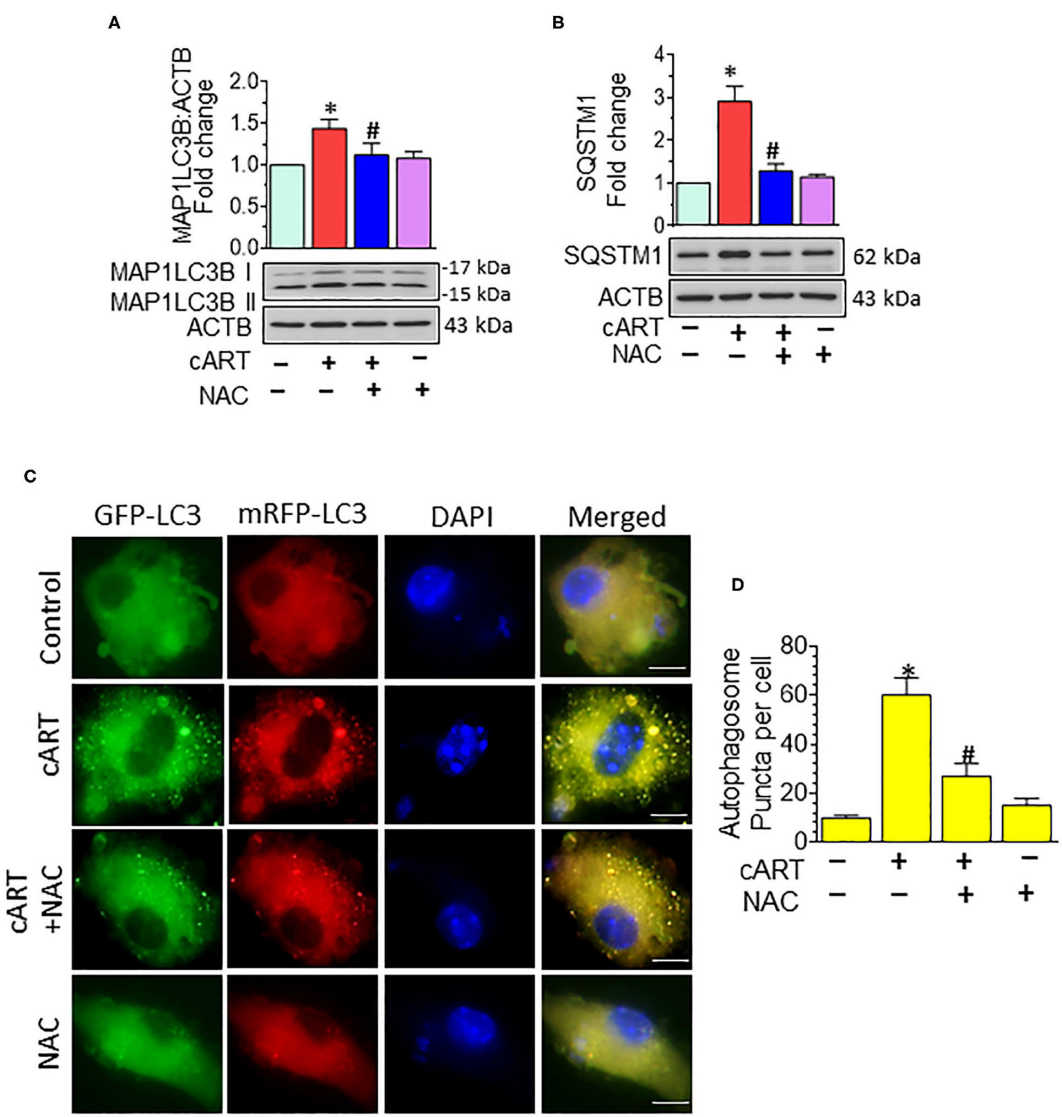
N-acetylcysteine (NAC) reverses combined antiretroviral therapy (cART)-mediated autophagy dysregulation in rat primary microglial cells (rPMs). **(A,B)** Representative Western blots showing treatment of NAC-reversed cART-mediated upregulation of autophagy markers microtubule-associated protein 1 light chain 3 beta (MAP1LC3B) **(A)** and sequestosome 1 (SQSTM1) **(B)**. **(C,D)** rPMs were seeded into 12-well plates followed by transfection of cells with the tandem fluorescent-tagged MAP1LC3B plasmid. Cells were then exposed to various treatments for an additional 24 h and fluorescent intensity assessed by confocal microscopy (Scale bar: 5 μm). A minimum of 50 randomly chosen cells for each experimental group were analyzed. For all Western blots, ACTB served as a protein loading control. Data are from three independent experiments and are expressed as means ± SEM and were analyzed using a one-way analysis of variance followed by a Bonferroni (Dunn) comparison of groups. *P < 0.05 vs. control; ^#^*P* < 0.05 vs. cART.

In our previous published study, we have shown cART-mediated autophagy dysregulation (13). Autophagy is regulated by autophagy-associated proteins, such as BECN1, an autophagosome initiation marker, and microtubule-associated protein 1 light chain 3 beta (MAP1LC3B/LC3B), an autophagosome formation marker, and SQSTM1/p62, an autophagy degradation marker. Defective or impaired autophagy, on the other hand, is shown to be associated with the accumulation of the protein p62 (46, 47). Data from our previous study showed no significant difference in the accumulation of MAP1LC3B and SQSTM1 in the rPMs exposed to cART in the presence/absence of Bafilomycin A1, which confirmed the accumulation of autophagosomes in the cART-treated rPMs. In the present study, we sought to determine the role of cART-mediated ROS induction in dysregulation of autophagy. To validate this, rPMs were treated with cART cocktail with or without NAC for 24 h, after which the expression of autophagy markers MAP1LC3B and SQSTM1 was assessed by Western blotting. As expected, and as shown in Figures 3A,B, the treatment of rPMs with NAC markedly blocked the cART-mediated upregulation of MAP1LC3B along with SQSTM1, thereby implying increased autophagosome–lysosome fusion. To further validate these findings and to decipher the ability of cART to regulate the autophagosome–lysosome fusion efficiency, rPMs were transfected with a tandem fluorescent-tagged MAP1LC3B reporter plasmid followed by treatment of cells with cART with or without NAC. This reporter plasmid is an indicator of the extent of autophagic flux, as evidenced by the fluorescent color (yellow or red) (31). Under basal conditions, there is an even distribution of the red and the green signals. Autophagosome formation is represented by yellow puncta, owing to the colocalization of red and green fluorescence. Additionally, the maturation stage is represented by the red puncta, as green fluorescence is quenched in the autolysosomes, owing to the acidic environment. As shown in Figures 3C,D, rPMs treated with cART exhibited a significant increase in the yellow puncta and decreased in the red puncta, thereby indicating incomplete autophagosome maturation. On the other hand, rPMs treated with NAC followed by cART exposure (for 24 h) demonstrated a significant increase in the red puncta with a moderate level of yellow puncta compared with rPMs exposed to cART alone.

### N-Acetylcysteine-Reversed Combined Antiretroviral Therapy-Mediated Activation of Rat Primary Microglial Cells

**Figure 4:**
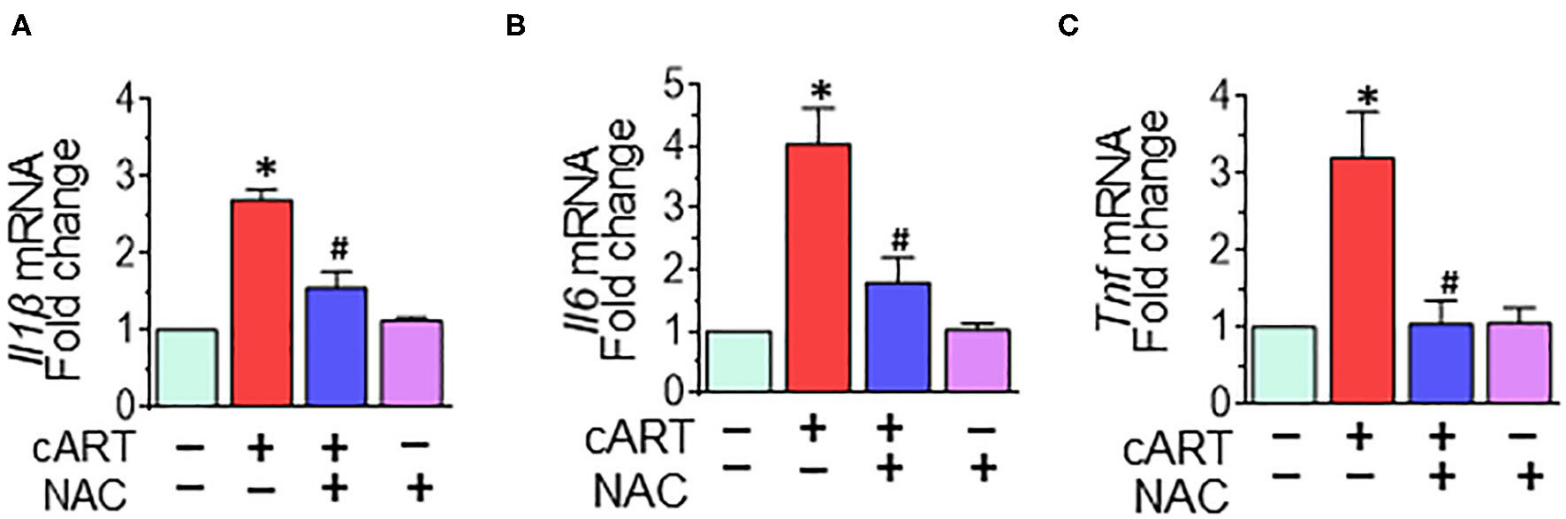
N-acetylcysteine (NAC) reverses combined antiretroviral therapy (cART)-mediated activation of rat primary microglial cells (rPMs). rPMs were seeded into six-well plates and subject to various treatments for 12 h. **(A–C)** Representative bar graph demonstrating NAC-mediated abrogation of cART-induced messenger RNA expression of pro-inflammatory cytokines: interleukin 1 beta (*Il1*β), interleukin 6 (*Il6*), and tumor necrosis factor (*Tnf*). Data are from three independent experiments and are represented as means ± SEM and were analyzed using one-way ANOVA. *P < 0.05 vs. control; ^#^*P* < 0.05 vs. cART.

Our previous findings demonstrated that in rPMs, cART treatment resulted in increased expression of pro-inflammatory cytokine messenger RNAs (mRNAs) (*Il1*β, *Il6*, and *Tnf* at 6- and 12-h post-treatment (13). Next, we sought to examine the role of NAC in abrogating cART-mediated activation of microglia. To validate this, rPMs were treated with the cART cocktail (TDV, FTC, DTG, each at 5 μM) with or without NAC (5 mM) for 12 h, after which, the expression of pro-inflammatory cytokine mRNAs was assessed by real-time quantitative PCR. Treatment of rPMs with NAC significantly blocked the cART-mediated upregulation of pro-inflammatory cytokine (*Il1*β, *Il6*, and *Tnf*) mRNAs (Figure 4).

### N-Acetylcysteine-Reversed Combined Antiretroviral Therapy-Mediated Lysosome Impairment and Autophagy Dysregulation *in vivo*

**Figure 5:**
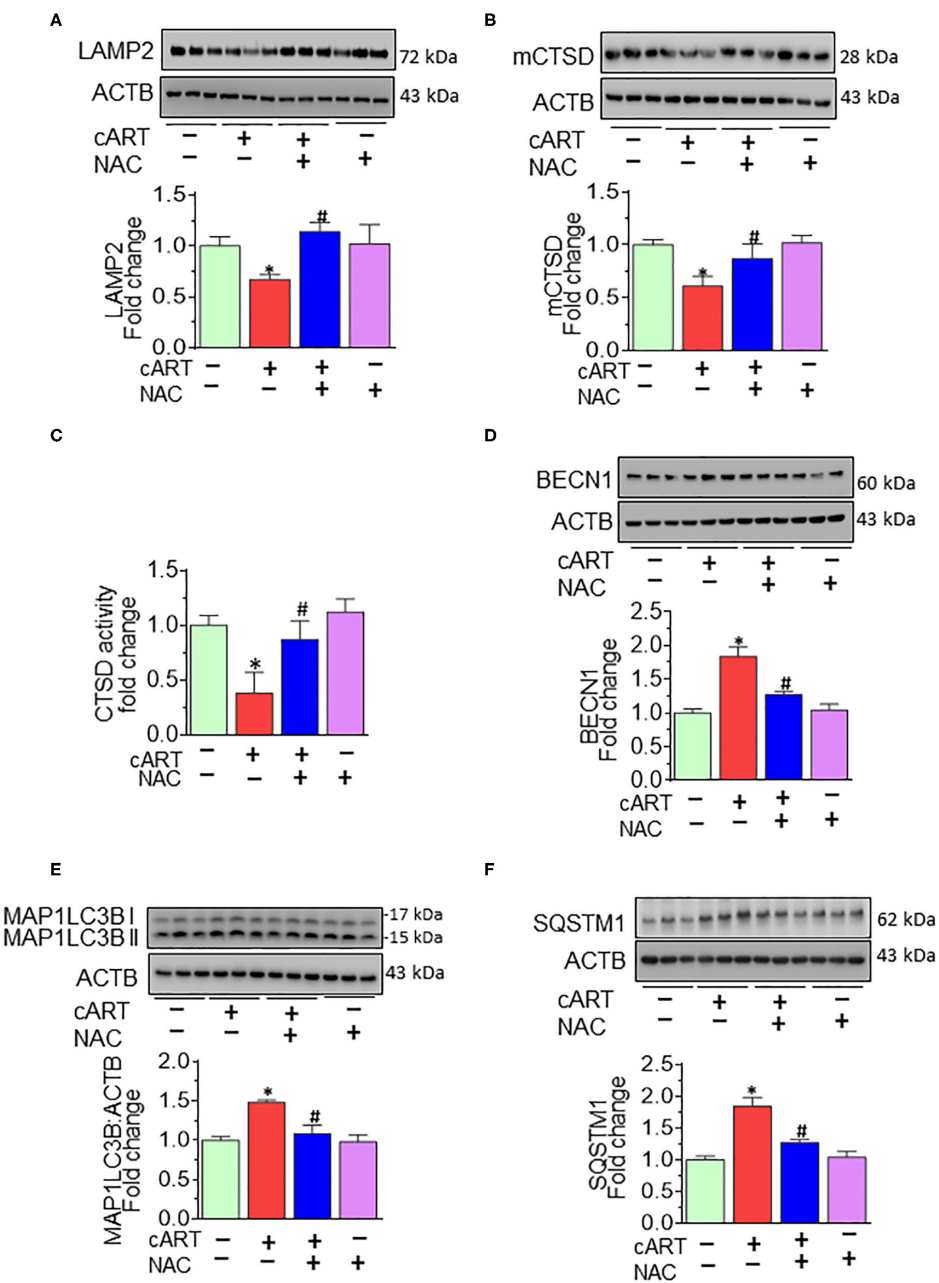
N-acetylcysteine (NAC) reverses combined antiretroviral therapy (cART)-mediated lysosome impairment and autophagy dysregulation *in vivo*. HIV Tg rats received cART injection with or without NAC (200 mg/kg) treatment (*n* = 3/group, intraperitoneal, 3 weeks). Saline-injected HIV Tg rats were used as controls. Protein homogenates of prefrontal cortices were prepared to detect the levels of indicated molecules. **(A,B)** Representative Western blots showing treatment of NAC-reversed cART-mediated downregulation of both LAMP2 **(A)** and mCTSD **(B)** in the prefrontal cortices. **(C)** Representative bar graph showing NAC-reversed cART-mediated downregulation of CTSD activity. **(D–F)** Representative Western blots showing treatment of NAC-reversed cART-mediated upregulation of beclin 1 (BECN1) **(D)**, microtubule-associated protein 1 light chain 3 beta (MAP1LC3B) **(E)**, and sequestosome 1 (SQSTM1) **(F)** in the prefrontal cortices of HIV Tg rats. For all Western blots, ACTB served as a protein loading control. Data are from three independent experiments and are represented as means ± SEM and were analyzed using one-way ANOVA. *P < 0.05 vs. control; ^#^*P* < 0.05 vs. cART.

Having demonstrated the protective effects of NAC on cART-mediated lysosomal damage and microglial activation *in vitro*, the next step was to explore *in vivo* the effects of cART on lysosomes and microglia. It must be noted that despite low viremia, viral proteins, such as transactivator of transcription (Tat) and the envelope gp120 do persist in the brains of cART-treated infected individuals (48). Therefore, to understand the efficacy of NAC in the context of cART, we resorted to using the well-established HIV Tg rat model. These rats express seven of nine HIV proteins with no active HIV replication *in vivo*, thereby best-mimicking a scenario of HIV-infected individuals on cART regimen, wherein the viral replication is often below the threshold of detection, although viral proteins continue to persist (49–52). Groups of HIV Tg rats were saline, cART, cART with NAC, and NAC-alone group, daily administration (intraperitoneal) for 3 weeks. Animals were then killed and brains removed for assessment of lysosomal markers and functioning and microglial status. Specifically, the prefrontal cortex region that is intricately linked with movement and cognitive decline in HAND patients was chosen for this study. Interestingly, similar to cell culture findings, HIV Tg rats exposed to cART also demonstrated the decreased expression of brain LAMP2, as well as mCTSD, and NAC treatment blocked these effects of cART (Figures 5A,B). The protective role of NAC against cART was also observed in lysosomal functioning, as evidenced by increased CTSD activity (Figure 5C) in the prefrontal cortex of NAC-treated rats compared with that in the cART-alone group.

We next sought to explore the effects of NAC treatment on cART-mediated dysregulation of autophagy in HIV Tg rats. Autophagy is regulated by autophagy-specific proteins, such as BECN1, MAP1LC3B/LC3B, and SQSTM1/p62. BECN1 acts during the initiation stage of autophagy by forming the isolation membrane, a double-membrane structure that engulfs cytoplasmic material to form the autophagosome. MAP1LC3B/LC3B is an autophagosome formation marker, and SQSTM1/p62 is an autophagy degradation marker. Defective or impaired autophagy is associated with the accumulation of p62 (46, 47). As shown in Figures 5D–F, and as expected, cART treatment significantly increased the expression of BECN1 (Figure 5D), MAP1LC3B (Figure 5E), and SQSTM1 (Figure 5F) in the prefrontal cortices of HIV Tg rats compared with rats not exposed to cART. Interestingly, NAC treatment blocked cART-mediated dysregulation of autophagy in the prefrontal cortices of HIV Tg rats.

### N-Acetylcysteine-Reversed Combined Antiretroviral Therapy-Mediated Microglial Activation *in vivo*

**Figure 6:**
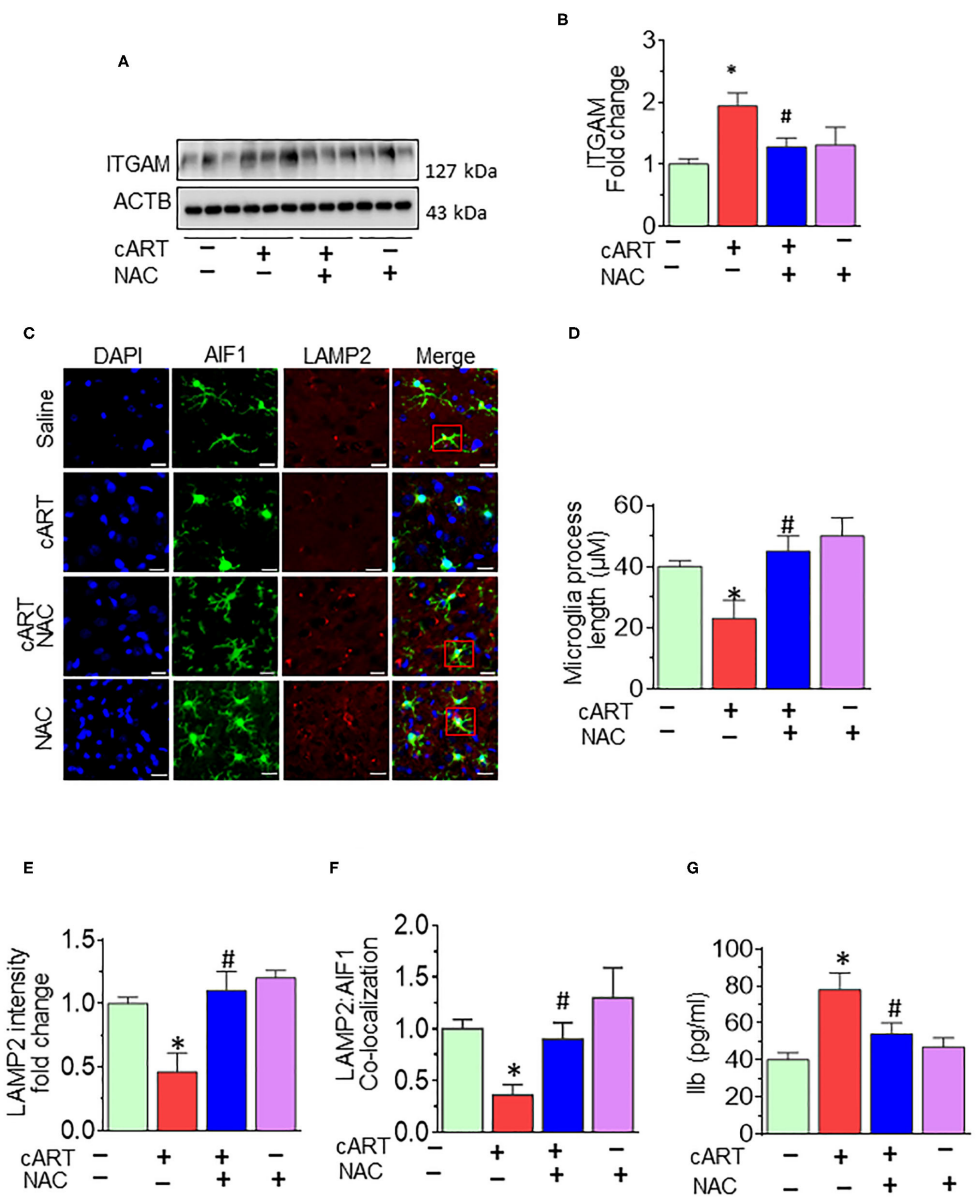
N-acetylcysteine (NAC) reverses combined antiretroviral therapy (cART)-mediated microglial activation *in vivo*. **(A,B)** Representative Western blot and bar graph showing treatment of NAC-reversed cART-mediated upregulation of integrin subunit alpha M (ITGAM) in the prefrontal cortices of HIV Tg rats. **(C)** Representative fluorescent-microscopic image showing the fluorescent intensity of allograft inflammatory factor 1 (AIF1) and lysosome-associated membrane protein 2 (LAMP2) in the prefrontal cortices of HIV Tg rats receiving various treatments (Scale bar: 20 μm). A minimum of 50 randomly chosen cells for each experimental group were analyzed. Red boxes in control, NAC + cART, and NAC-alone groups represent the microglial cells with ramified cellular processes with LAMP2 colocalization, which is absent in the cART group. **(D)** Representative bar graph showing treatment of NAC-reversed cART-mediated downregulation of microglial process length. **(E,F)** Representative bar graphs showing treatment of NAC-reversed cART-mediated downregulation of LAMP2 staining and decreases colocalization of LAMP2 and AIF1 in microglia in the prefrontal cortex region. **(G)** Representative bar graph showing treatment of NAC-reversed cART-mediated upregulation of Il1β protein levels in the prefrontal cortex. Data are from three independent experiments and are represented as means ± SEM and were analyzed using one-way ANOVA. *P < 0.05 vs. control; ^#^*P* < 0.05 vs. cART.

As shown in Figures 6A,B, cART administration resulted in significantly increased expression of microglial marker—ITGAM—in the prefrontal cortex, and these effects were abrogated in rats treated with NAC. To further validate lysosomal alterations in microglia *in vivo*, we next performed double immunostaining on sections of the prefrontal cortex from HIV Tg rats exposed to NAC/saline and cART. Exposure of cART to HIV Tg rats resulted in increased expression of microglial activation marker—AIF1 (Figure S5) with a concomitant decrease in the length of microglial processes (Figures 6C,D). HIV Tg rats exposed to cART also exhibited decreased LAMP2 expression and decreased colocalization of LAMP2 with AIF1 (Figures 6E,F) in the prefrontal cortices. As expected, NAC treatment abrogated cART-mediated impairment of lysosomes and microglial activation. The protective role of NAC was also observed in neuroinflammatory responses, as evidenced by decreased Il1β protein levels (Figure 6G) in the prefrontal cortices of HIV Tg rats treated with cART with NAC compared with the cART-alone group. Furthermore, we have checked the neuronal marker microtubule-associated protein 2 (MAP2) in the prefrontal cortex of HIV Tg rats treated with cART with or without NAC. Figure S4 shows decreased MAP2 staining in the prefrontal cortex of the HIV Tg rats treated with cART, which was reversed by NAC treatment. MAP2 stabilizes neuronal shape by promoting microtubule synthesis and cross-linking with other components of the cytoskeleton and regulating microtubule networks in the axons and dendrites of neurons (53). Studies have reported that prolonged LMP activates lysosomal-dependent cell death. To exclude cell death-induced activation of microglia, we have also examined the expression of the apoptotic marker, CASP3, in the brains of HIV Tg rats treated with cART. As shown in Figure S2, there was no significant change in cleaved CASP3 expression in HIV Tg rats in the presence or absence of cART.

## Discussion

Increased neuroinflammation and microglia activation are hallmark features of HIV-infected individuals on cART (2, 6, 7). Mechanism(s) underlying these processes, although extensively studied, remains less understood. It has been well-documented that despite the effectiveness of cART in suppressing viremia, the long-term use of cART could result in severe adverse effects, including oxidative stress, mitochondrial damage, disruption of phagocytosis, and amyloid-β production in various cells (54–59). Induction of oxidative stress has been widely reported in the setting of HIV-1 infection and antiretroviral therapy (20, 21, 35). An oxidative imbalance has been demonstrated in the serum of HIV-1-infected individuals receiving antiretroviral therapy (21). There are several combinations of antiretrovirals that are clinically used to treat HIV infection (60). In the present study, we demonstrated that exposure of microglia to the three commonly used antiretrovirals (TDF, FTC, and DTG) induced the generation of ROS, which, in turn, impaired lysosomal functioning and blocked autophagosome–lysosome fusion, ultimately resulting in microglial activation.

Lysosomes are specialized cellular organelles critical for the degradation of macromolecules/damaged organelles. Lysosomal dysfunction is shown to correlate with inflammation (14, 15). Various studies have demonstrated the role of HIV-1 Tat protein (61) as well as antiretrovirals (38) in mediating endolysosomal dysfunction. Findings from our earlier *in vitro* study demonstrated that cART-mediated activation of microglia involved impaired lysosomal functioning and dysregulated autophagy (13). Herein, we sought to determine the involvement of oxidative stress as an upstream event of cART-mediated lysosomal dysfunction, leading to microglial activation. We also examined the role of NAC in mitigating cART-mediated defects in microglia. Furthermore, our *in vitro* findings were also corroborated by *in vivo* studies, wherein we examined the protective effects of NAC against cART-mediated microglial activation in HIV Tg rats.

In the present study, we demonstrated that exposure of rPMs to cART resulted in the induction of ROS and that the ROS scavenger, NAC, significantly abrogated cART-mediated induction of ROS. NAC not only scavenges ROS but also increases the concentration of intracellular GSH to reduce oxidative stress (40). Studies have shown GSH deficiency in HIV-infected individuals, which was replenished by NAC treatment (41). Another study showed oxidative stress-mediated blood–brain barrier damage in mice exposed to HIV proteins (gp120 and Tat) and methamphetamine, which was protected by N-acetylcysteine amide, a modified form of NAC (62). NAC is an inexpensive generic supplemental drug and one of the 40 essential medicines in the list laid out by the World Health Organization (40). NAC is used as an adjunctive therapeutics for several neurological and neuropsychiatric disorders (63, 64).

Our previously published report has demonstrated cART-mediated lysosomal dysfunction in rPMs (13). Herein, we sought to examine the protective effects of NAC treatment on cART-mediated lysosomal dysfunction. Our findings demonstrated that exposure of rPMs to cART resulted in impaired lysosomes, as shown by downregulated expression of LAMP2 and mCTSD, and this effect was abrogated by treatment of cells with NAC. Next, we also sought to determine the effects of NAC treatment on cART-mediated defects in lysosomal functions. For this, we examined LMP, CTSD activity, and lysosomal pH. Treatment of NAC protected rPMs against cART-mediated increased LMP and decreased CTSD activity. NAC also maintained lysosomal pH in rPMs exposed to cART. LMP is the major cause of proton leakage through a destabilized membrane, resulting in loss of the pH gradient (65, 66). Changes in LMP were further confirmed by assessing the GAL3 translocation into the damaged lysosomes. Normally, GAL3 is uniformly distributed in the cells. Upon insult and after LMP, GAL3 translocates into the leaky lysosomes, resulting in the formation of puncta (45). In keeping with this, our studies demonstrated significant colocalization of LAGLS3 with the lysosomal LAMP2 in cART-exposed microglial cells. Intriguingly, NAC treatment abrogated translocation of GAL3 into the damaged lysosomes. These data further confirmed NAC-mediated protection of lysosomes in cART-exposed rPMs.

Lysosomes are critical for the maturation of autophagy, as the fusion of autophagosomes and lysosomes form the autolysosome, which is necessary for protein degradation (67, 68). The autophagy–lysosome pathway has been implicated in many disease conditions (69–72). Dysregulated autophagy is a hallmark feature of many types of cancers (67, 73) and multiple neurodegenerative diseases, including Parkinson's (74, 75), Alzheimer's diseases (76, 77), and HIV neuropathogenesis (78, 79).

Our findings also suggested cART-mediated dysregulation of autophagy as assessed by increased expression of autophagy mediators MAP1LC3B and SQSTM1 in rPMS exposed to cART. cART-mediated blockage of autophagy was assessed using tandem fluorescent-tagged MAP1LC3B plasmid *in vitro*. Intriguingly, ROS scavenger and lysosome protecting agent NAC ameliorated cART-mediated dysregulation of autophagy in microglia. Autophagy dysregulation and neuroinflammation are closely linked in the development of neurodegeneration (80). CNS Inflammation is a common feature in HIV-infected patients on cART (81). Our findings demonstrated cART-mediated upregulation of pro-inflammatory mediators, such as *Il6, Il1*β, and *Tnf*. Furthermore, the treatment of NAC inhibited cART-mediated upregulation of pro-inflammatory mediators in rPMs. Intriguingly, NAC ameliorated cART-mediated lysosomal dysfunction, autophagy dysregulation, and microglial activation, implying thereby that cART-mediated induction of ROS is upstream of lysosomal damage, dysregulated autophagy, and microglial activation. These results thus underscore the fact that strategies aimed at curbing ROS and protecting lysosomes could dampen cART-mediated neuroinflammation in treated HIV-infected individuals. NAC can thus be envisioned as an ideal candidate for scavenging cellular ROS and protecting the lysosomal membrane (66, 82). Although various studies have underscored the role of NAC as an adjunctive treatment in diseases involving ROS, the failure in clinical trials to prove the beneficial effects of antioxidant therapies remains a great disappointment in the field. For example, clinical trials with high doses of NAC did not improve respiratory health in patients with COPD and chronic bronchitis (83). Furthermore, in a large randomized trial, NAC did not reduce the risk of acute kidney injury or other clinically relevant outcomes in patients undergoing coronary and peripheral vascular angiography (84, 85). However, the major reason for the clinical trial failure was premature suspension or termination of the studies (40). Moreover, adjunctive therapy using antioxidants is one of the best approaches to harness their beneficial effects as well as to diminish the likelihood of disease exacerbation. Unraveling the mechanisms of action, optimization of concentration, and delivery at appropriate physiological sites could aid in improving the treatment efficacy of antioxidants, thus making them as efficacious and successful therapeutic options. More clinical trials are underway that could underpin the role of NAC as an antioxidant (40, 86).

Current studies were done in isolated rat primary microglia, and we do acknowledge that data from *in vitro* microglial experiments should be interpreted with utmost care while extrapolating data in the context of the whole brain. Microglia activation status is continually affected by external cues. In cell culture, microglia are devoid of any inhibitory signals owing to lack of cell–cell interactions between microglia and other CNS cells, which are essential for maintaining microglia in quiescence (87). Furthermore, *in vitro* microglia are cultured in serum-containing medium, which is not the case under *in vivo* conditions. Serum is a poorly defined cell culture component with batch-to-batch variability that can contribute negatively to rigor and reproducibility (88, 89). Another challenge of working with microglial cultures is their species variability. Differential responses have been exhibited by rat and mouse microglial cells to the external stimuli (90). It has also been reported that human and mouse microglia age differently (91). Overall, it is a major challenge to work with dissociated microglia with the optimal mix of environmental cues that they receive *in vivo*. In the absence of other types of CNS cells, the phenotype of microglia in cultures begins to significantly diverge from that of microglia *in vivo*, leading, in turn, to a major limitation in their predictive ability for *in vivo* biology (92).

In the present study, we resorted to validating our cell culture findings in a rodent model that recapitulates some aspects of HAND—the HIV Tg rats. HIV Tg rat mimics many of the metabolic disturbances seen in HIV-1-infected humans and is a useful tool to study the relationship between the accumulation of HIV-1 protein in the brain and the manifested clinical neurological processes (49–52). Our findings demonstrated that similar to cell culture findings, exposure of HIV Tg rats to cART also resulted in increased activation of microglia, as evidenced by increased AIF1 density and decreased length of the microglial processes in the prefrontal cortices. Our findings also demonstrated lysosomal dysfunction and autophagy dysregulation in the prefrontal cortices of HIV Tg rats exposed to cART. Some reports show dysregulated autophagy in the prefrontal cortex of post-mortem brains of persons with HIV-1-associated encephalitis (93). There is also a report that products of SIV-infected microglia inhibit neuronal autophagy (94). Based on the premise that dysregulated autophagy and inflammation are implicated as the driving force underlying pathogenesis of HAND, our results underscore the role of cART as a contributor to the progression of HAND.

**Figure 7:**
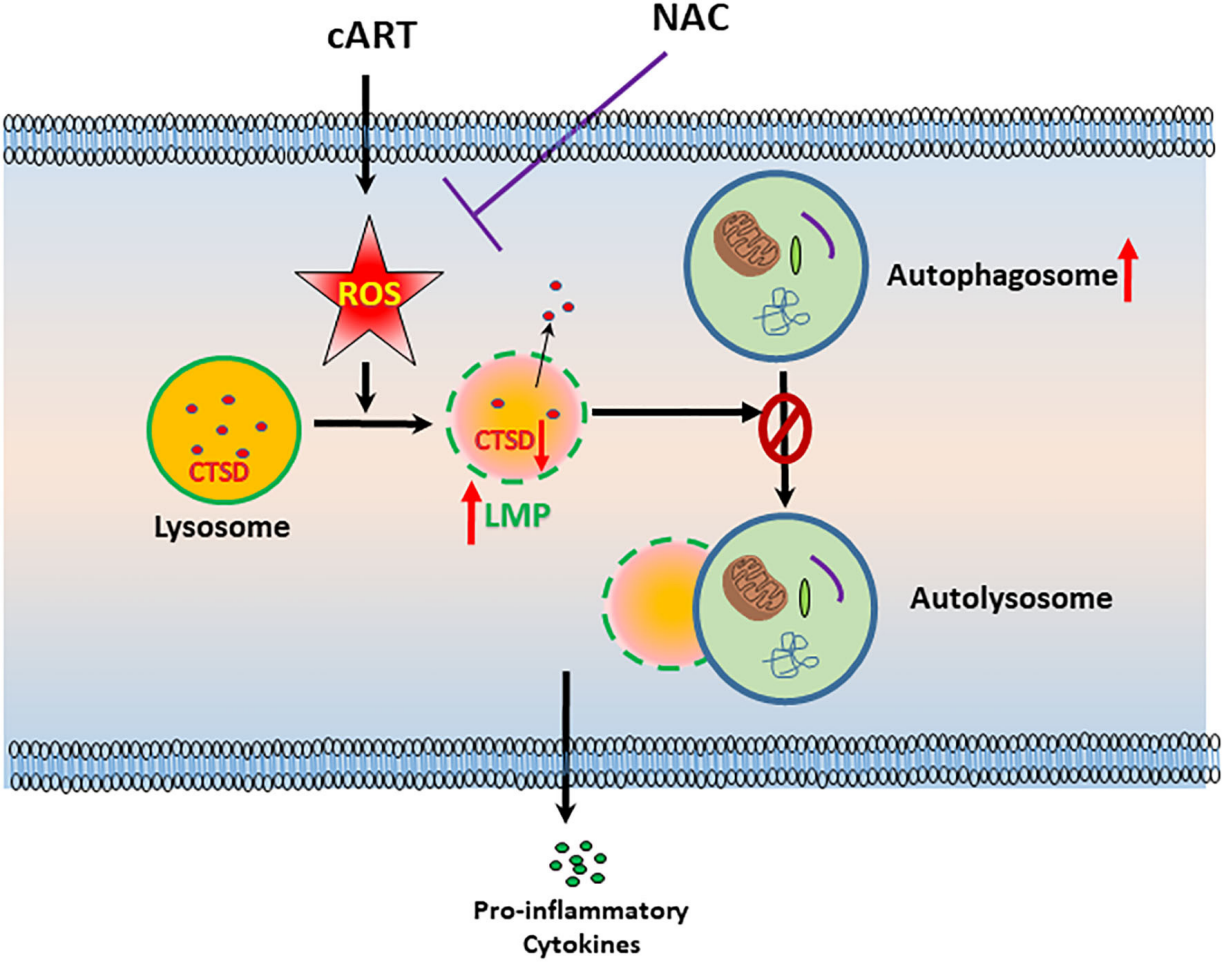
Schematic depicting the involvement of reactive oxygen species (ROS) in combined antiretroviral therapy (cART)-mediated autophagy–lysosomal dysfunction in microglia. Exposure of microglia to cART increases ROS generation, leading, in turn, lysosomal dysfunction and autophagy dysregulation, which ultimately led to microglial activation and increased expression of pro-inflammatory cytokines. ROS scavenger N-acetylcysteine (NAC) reversed these deleterious effects of cART.

In summary, our findings demonstrate that ROS-mediated autophagy–lysosomal dysfunction is central in cART-mediated microglial activation and that the potent ROS scavenger—NAC—reverses cART-mediated effects both *in vitro* and *in vivo* (Figure 7). Antioxidant-mediated mitigation of oxidative stress could be considered as an adjunctive therapeutic strategy for ameliorating/dampening some of the neurological complications of HAND.

## Data Availability Statement

The original contributions presented in the study are included in the article/Supplementary Material, further inquiries can be directed to the corresponding author/s.

## Ethics Statement

All animal procedures were performed according to the protocols approved by the Institutional Animal Care and Use Committee of the University of Nebraska Medical Center and the National Institutes of Health.

## Author Contributions

SB, ATr, and M-LG designed the experiments. ATr, ATh, EC, PP, MB, and FN performed the experiments. SB, ATr, and M-LG analyzed the data and wrote the manuscript. All authors contributed to the article and approved the submitted version.

## Conflict of Interest

The authors declare that the research was conducted in the absence of any commercial or financial relationships that could be construed as a potential conflict of interest.

## Abbreviations

ACTB: actin, beta
AIF1: allograft inflammatory factor 1
BECN1: beclin 1, autophagy related
cART: combined antiretroviral therapy
CASP3: caspase 3
CNS: central nervous system
CTSD: cathepsin D
DAPI: 4,6-diamidino-2-phenylindole
DMEM: Dulbecco modified Eagle medium
DTG: dolutegravir
FBS: fetal bovine serum
FTC: emtricitabine
GAPDH: glyceraldehyde-3-phosphate dehydrogenase
HAND: HIV-1-associated neurocognitive disorders
HIV-1 Tat: human immunodeficiency virus-1 transactivator of transcription
*Il1*β, interleukin 1: beta
*Il6*: interleukin 6
ITGAM: integrin subunit alpha M
LAMP2: lysosome-associated membrane protein 2
GAL3: galectin 3
LMP: lysosomal membrane permeabilization
MAP1LC3B: microtubule-associated protein 1 light chain 3 beta
mitoTEMPO: (2-(2,2,6,6-Tetramethylpiperidin-1-oxyl-4-ylamino)-2-oxoethyl)triphenylphosphonium chloride
NAC: N-acetylcysteine
NRTIs: nucleoside reverse transcriptase inhibitors
PBS: phosphate-buffered saline
ROS: reactive oxygen species
rPMs: rat primary microglial cells
SQSTM1: sequestosome 1
TBHP: tert-butyl hydroperoxide
TDF: tenofovir disoproxil fumarate
TEMPOL: 4-hydroxy-2,2,6,6-tetramethylpiperidin-1-oxyl
*Tnf*: tumor necrosis factor.
