# Supplementary material for: N-Acetylcysteine Reverses Antiretroviral-Mediated Microglial Activation by Attenuating Autophagy-Lysosomal Dysfunction
Source: Front Neurol. 2020 Sep 4;11:840. doi: 10.3389/fneur.2020.00840 (PMC7498983; doi:10.3389/fneur.2020.00840)
Supplement: Supplementary file 1 [file Presentation_1.PPTX]

## Slide 1
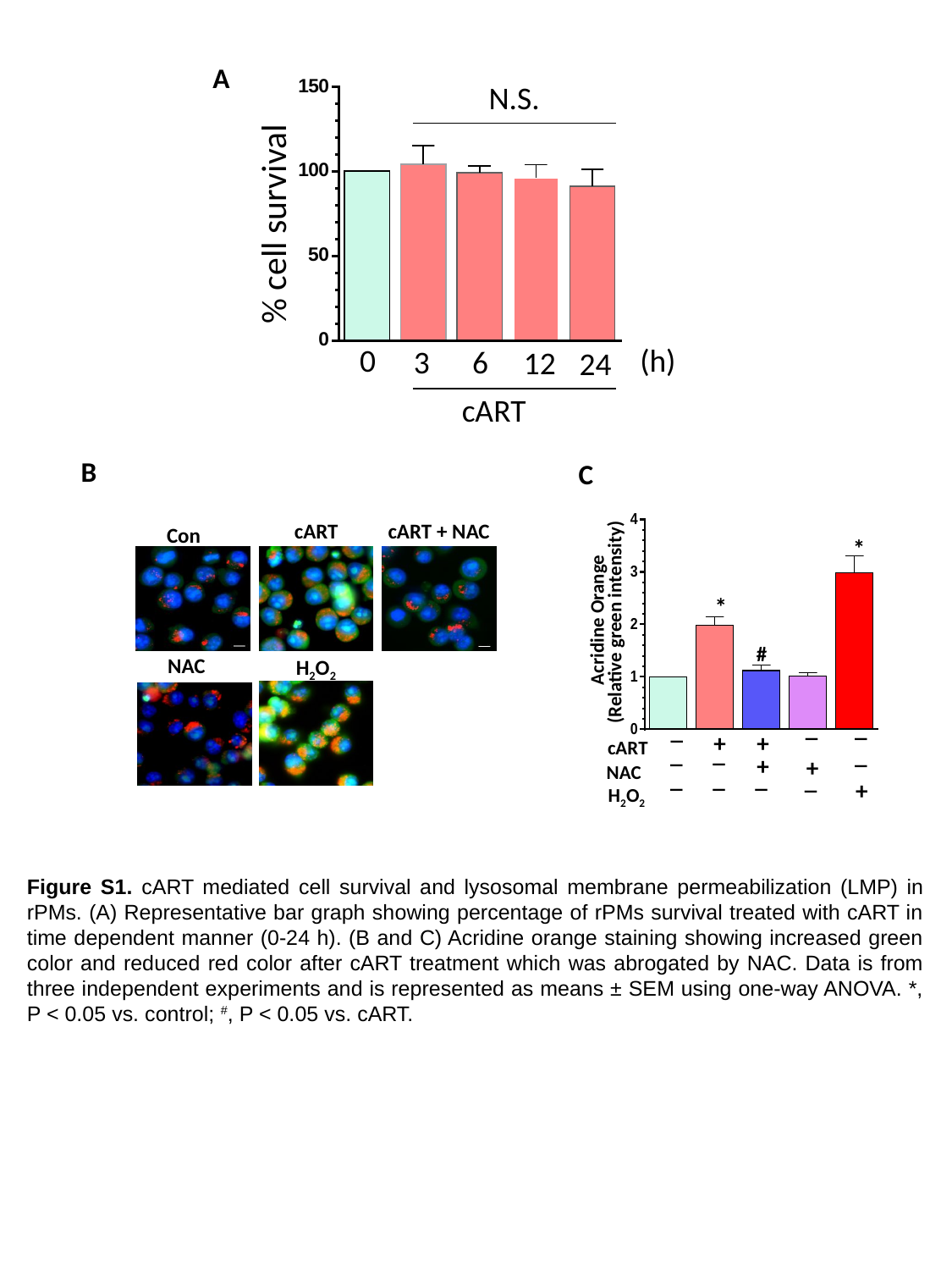

N.S.
% cell survival
0
(h)
3
6
12
24
cART
A
B
C
*
*
Acridine Orange
(Relative green intensity)
#
_
_
_
+
+
_
_
_
cART
+
+
_
_
_
_
NAC
+
H2O2
cART
cART + NAC
Con
NAC
H2O2
Figure S1. cART mediated cell survival and lysosomal membrane permeabilization (LMP) in rPMs. (A) Representative bar graph showing percentage of rPMs survival treated with cART in time dependent manner (0-24 h). (B and C) Acridine orange staining showing increased green color and reduced red color after cART treatment which was abrogated by NAC. Data is from three independent experiments and is represented as means ± SEM using one-way ANOVA. *, P < 0.05 vs. control; #, P < 0.05 vs. cART.

## Slide 2
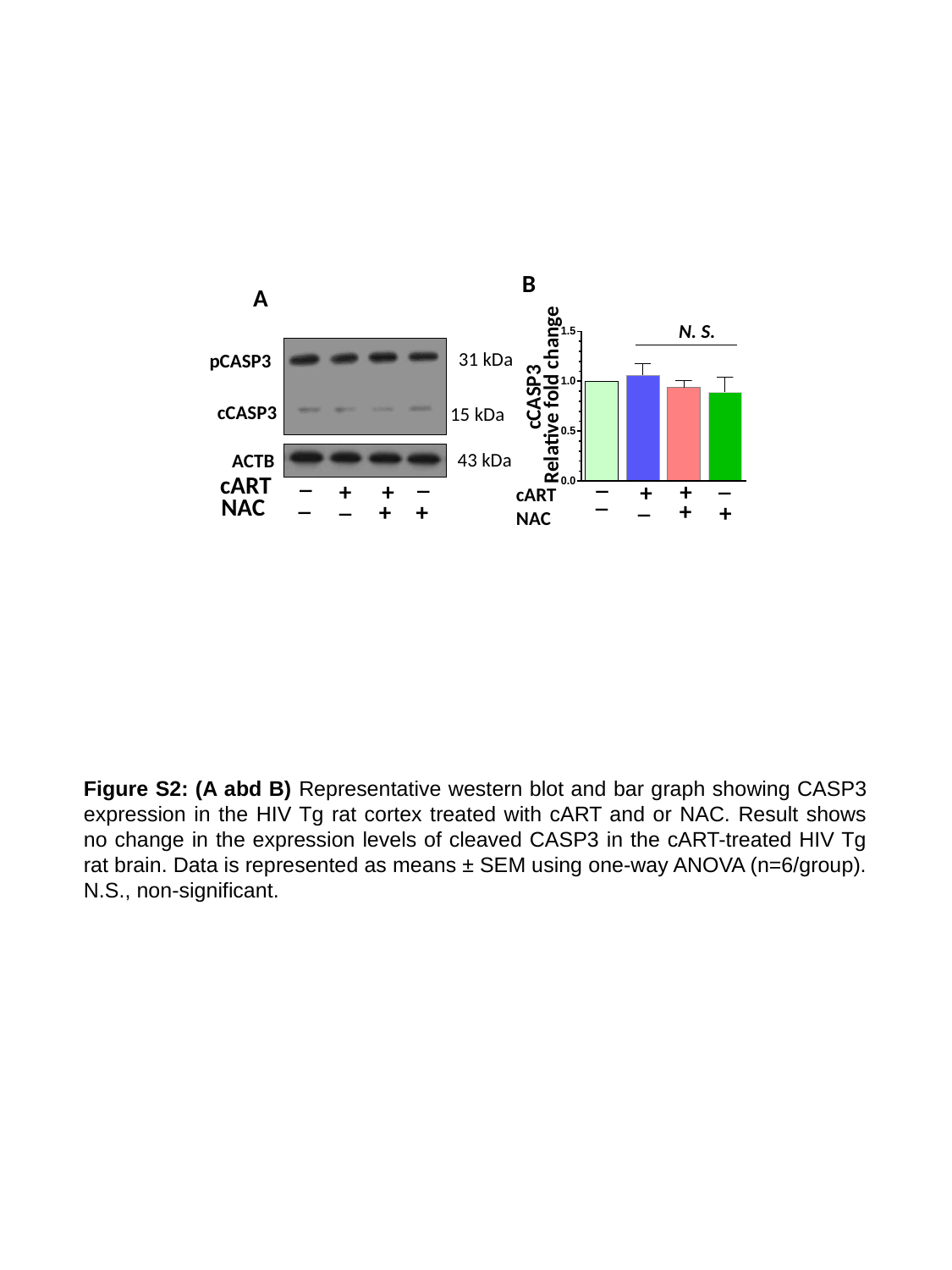

B
A
cCASP3
Relative fold change
_
_
+
+
cART
NAC
_
_
+
+
N. S.
31 kDa
pCASP3
cCASP3
15 kDa
43 kDa
ACTB
_
_
+
+
cART
_
_
+
+
NAC
Figure S2: (A abd B) Representative western blot and bar graph showing CASP3 expression in the HIV Tg rat cortex treated with cART and or NAC. Result shows no change in the expression levels of cleaved CASP3 in the cART-treated HIV Tg rat brain. Data is represented as means ± SEM using one-way ANOVA (n=6/group). N.S., non-significant.

## Slide 3
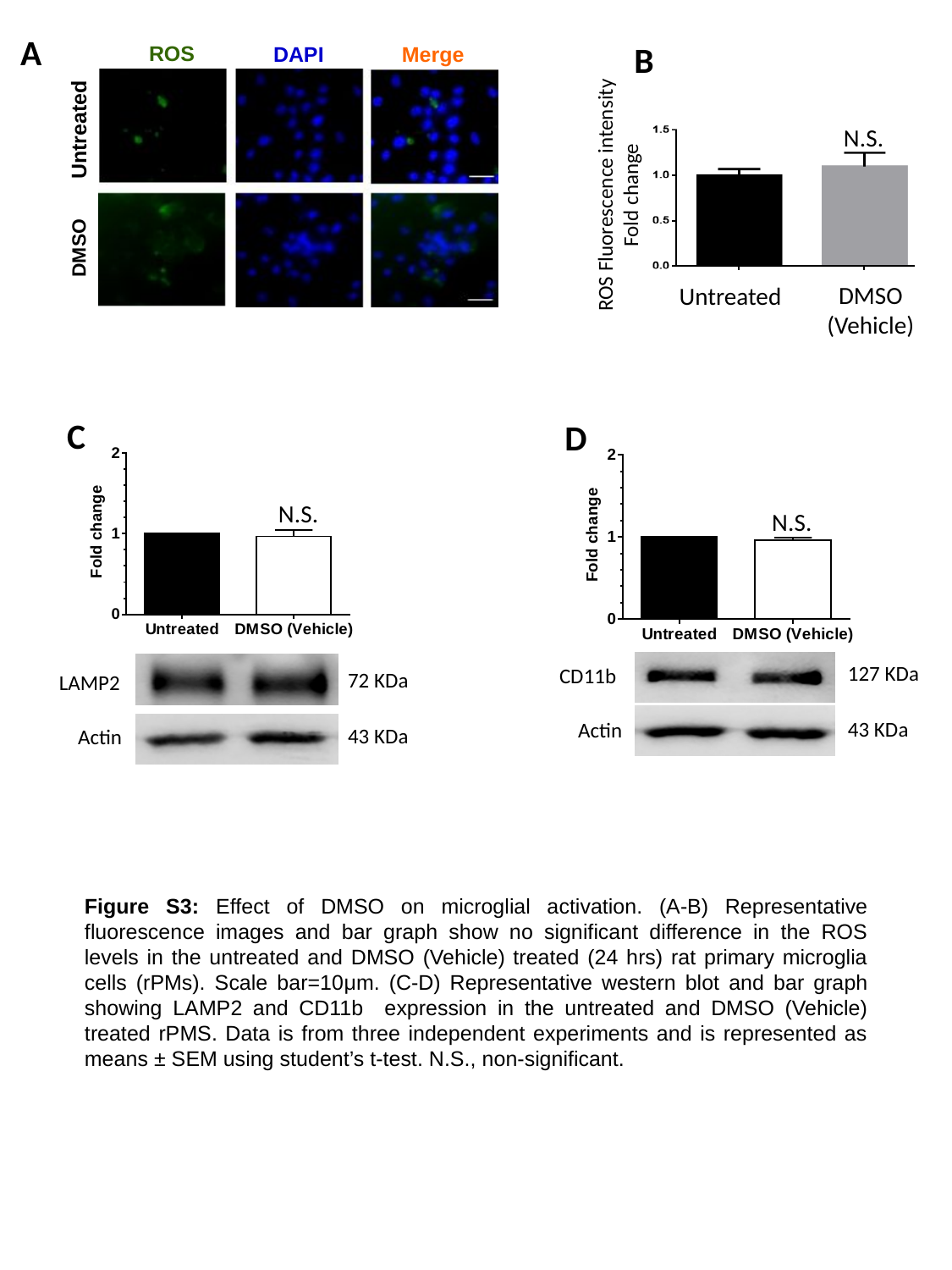

A
B
ROS
DAPI
Merge
Untreated
N.S.
ROS Fluorescence intensity
Fold change
DMSO (Vehicle)
Untreated
DMSO
C
D
N.S.
N.S.
127 KDa
CD11b
72 KDa
LAMP2
43 KDa
Actin
43 KDa
Actin
Figure S3: Effect of DMSO on microglial activation. (A-B) Representative fluorescence images and bar graph show no significant difference in the ROS levels in the untreated and DMSO (Vehicle) treated (24 hrs) rat primary microglia cells (rPMs). Scale bar=10μm. (C-D) Representative western blot and bar graph showing LAMP2 and CD11b expression in the untreated and DMSO (Vehicle) treated rPMS. Data is from three independent experiments and is represented as means ± SEM using student’s t-test. N.S., non-significant.

## Slide 4
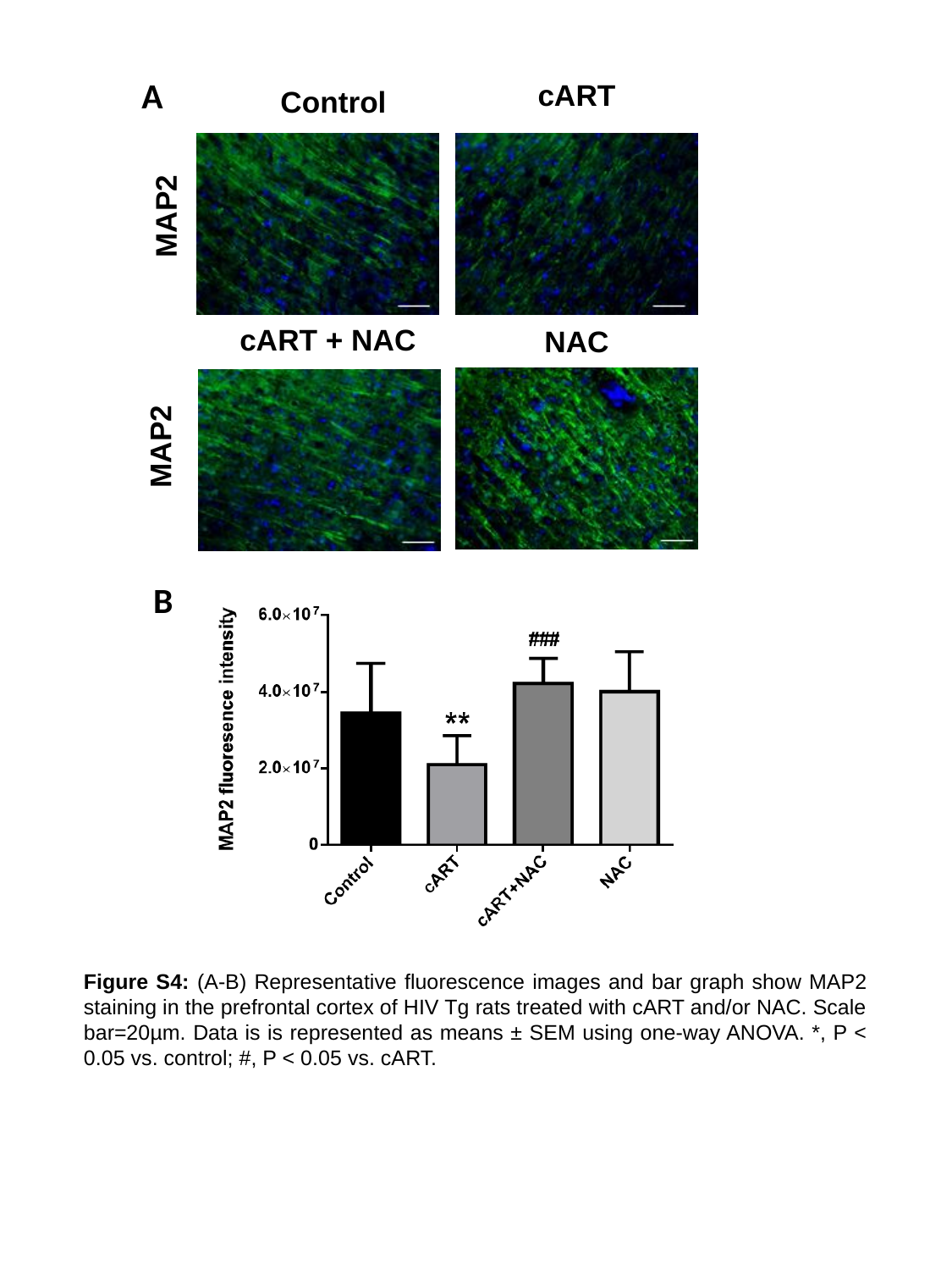

cART
Control
A
MAP2
cART + NAC
NAC
MAP2
B
Figure S4: (A-B) Representative fluorescence images and bar graph show MAP2 staining in the prefrontal cortex of HIV Tg rats treated with cART and/or NAC. Scale bar=20µm. Data is is represented as means ± SEM using one-way ANOVA. *, P < 0.05 vs. control; #, P < 0.05 vs. cART.

## Slide 5
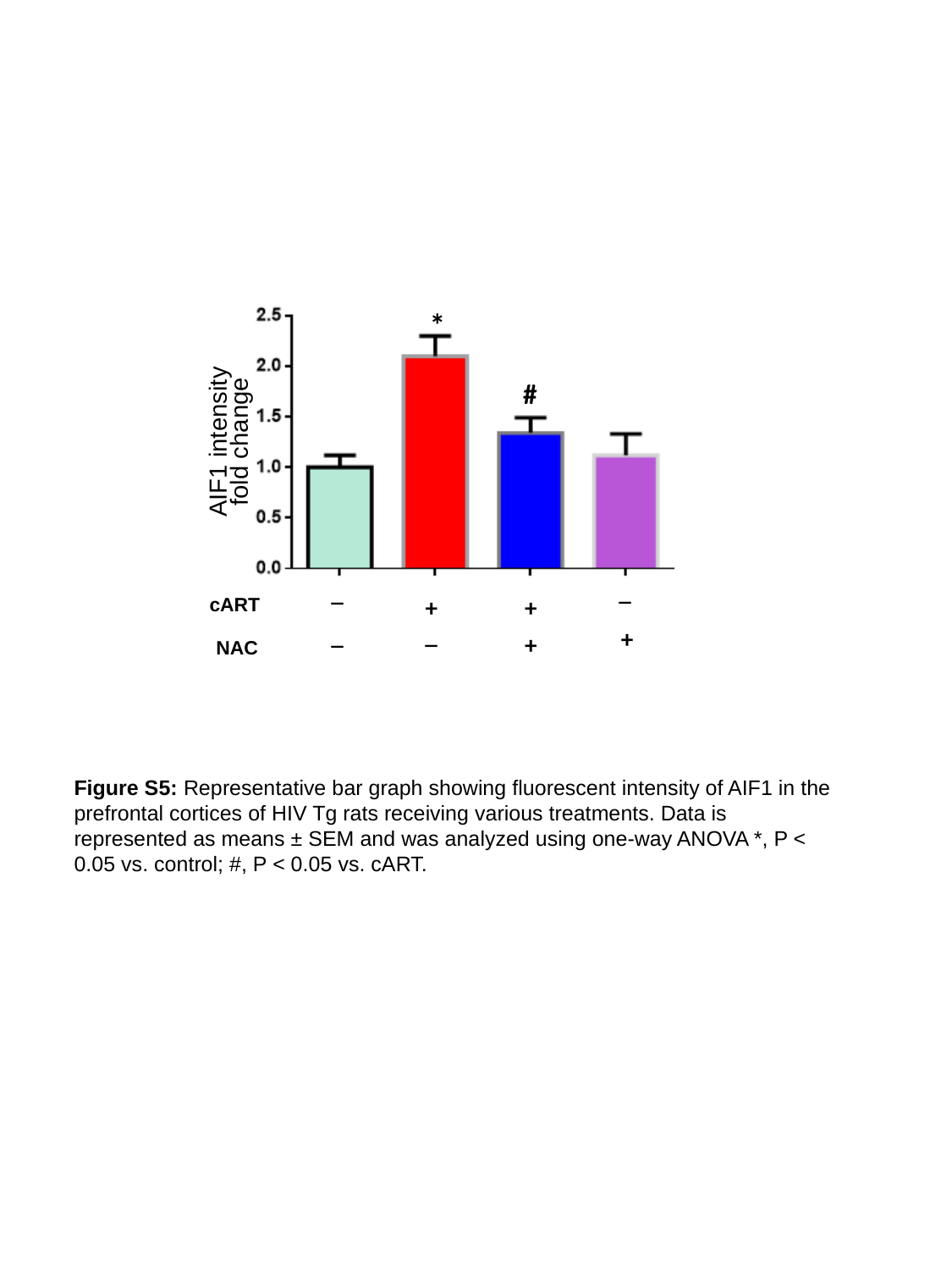

*
#
AIF1 intensity
fold change
_
_
+
+
cART
_
_
+
+
NAC
Figure S5: Representative bar graph showing fluorescent intensity of AIF1 in the prefrontal cortices of HIV Tg rats receiving various treatments. Data is represented as means ± SEM and was analyzed using one-way ANOVA *, P < 0.05 vs. control; #, P < 0.05 vs. cART.
